# Civilian Practitioners and Military Hospitals

**Published:** 1917-09-29

**Authors:** 


### CIVILIAN PRACTITIONERS AND MILITARY HOSPITALS.

The official case for the abrupt discharge of civil practitioners who have been giving part-time service in the military hospitals has hardly been bettered by its apologists. Anxiety to preserve the medical interests of the civilian community is an excellent sentiment, and must be recognised as a welcome, if somewhat tardy, sign of grace. But a more fruitful application of it would have been secured had the authorities consulted the Medical War Committees, who know exactly where the release of medical practitioners from military duties would have been helpful and where, on the other hand, such release was not of value. To put into the mouth of the Central Medical War Committee the statement that "if you can release some part-time men for civil practice, we can give you more men for the Army" is hardly cricket, for this even "in effect" is not what the Committee said. We fancy that the Committee did say something about the necessity of employing more effectively the practitioners who are already in the R.A.M.C., and the official appointment of a committee of inquiry on this point would seem to justify the suggestion as having at least a *prima facie* value. When the Army organisation of the doctors has been searched with the same scrutiny as that which has been applied to the civilian profession we shall know where economies can be practised, and then will be the reasonable time to make such rearrangements as this knowledge dictates. The civilian profession is far removed from any desire to set up a division between themselves and their military colleagues, and, indeed, such a division, unnatural enough at any time, would be absurd just now when some nine-tenths of the R.A.M.C. is formed by men who but yesterday were in civilian practice. Again, it is said that the employment of part-time men is uneconomical if—and there is much virtue in the word—full-time men are available. Whether the financial claim is well founded or not need not now be discussed, but the "economy" of medical service has to take count of factors other than mere price. Even if full-time men are "available" some question as to their experience in relation to that of the men they succeed must be a matter of concern not only to authorities who dwell securely in comfortable offices but still more to wounded soldiers dependent on the surgical skill which is imposed on them. Finally, we learn that the great need of the hour is for orthopædic surgeons, and the newcomers, it appears to be suggested, are all or mainly of this order. Really if nothing better than this could be said a stern official silence would have been a more prudent policy.

We are glad to learn that "an error in the cables" is really responsible for the misunderstanding, for, of course, it would be unjustifiable to expect discretion and courtesy from these useful but mechanical servants of the State. Further, the general sweep at first intimated is not, it seems, to be carried out, and the wounded are not abruptly to lose the skilled help of practitioners who have served them—and the country—efficiently during the last three years. Another evidence of repentance is the dispatch of a suitable letter of thanks to the doctors concerned, not a few of whom resent the ungracious character of the earlier communication. The whole incident emphasises a contention which has previously been urged—namely, that the military authorities of the Army would be well advised to seek to act in co-operation with the authorised representatives of the civilian profession. These are full of anxiety to help in every possible way, but it is useless to expect continued goodwill if men who have done yeoman service for the national cause are at one time utilised and at another ignored as happens to suit the official convenience. We trust the bitterness of the present incident will not prejudice future negotiations, but the lesson it announces must be duly learned.

